# Thiamine Deficiency With Mashed Potatoes: A Novel Case of Wernicke Encephalopathy Manifesting With Retinal Hemorrhages in a Pediatric Patient

**DOI:** 10.7759/cureus.10429

**Published:** 2020-09-13

**Authors:** Harmanpreet K Tiwana, Gerald Raymond, Kevin Littleton, Rishi Singhal, Ashutosh Kumar

**Affiliations:** 1 Neurology, Dartmouth-Hitchcock Medical Center, Lebanon, USA; 2 Genetics, Johns Hopkins University School of Medicine, Baltimore, USA; 3 Neurology, University of California Davis, Davis, USA; 4 Ophthalmology, Ohio State University Medical Sciences, Columbus, USA; 5 Pediatric Neurology, Penn State Health Milton S. Hershey Medical Center, Hershey, USA

**Keywords:** thiamine, wernicke encephalopathy, delirium, diplopia

## Abstract

We present a case report of a 17-year-old obese female with one-week onset of progressive confusion and double vision, associated with 50-pound weight loss in five months. On fundus examination, retinal hemorrhages and abnormalities of eye movements were seen. MRI showed abnormalities that were consistent with diagnosis of Wernicke encephalopathy (WE). Thiamine replacement caused gradual improvement in patient’s symptoms. Peripapillary hemorrhages on fundus examination, as seen in our patient, have been rarely seen in WE. Obesity with retinal hemorrhages, diplopia, etc. can be misleading due to idiopathic intracranial hypertension being the most common presentation in this subset. Thus, fundoscopy should be part of routine examination in WE-suspected patients.

## Introduction

Wernicke encephalopathy (WE) is a neuropsychiatric condition due to deficiency of vitamin B1 (thiamine) [1]. It consists of triad of restricted eye movements, delirium, and gait abnormalities. Commonly seen in adult patients with chronic alcoholism, it may also be seen with poor nutrition or impaired absorption from umpteen causes. We are presenting this case as WE is considered an uncommon diagnosis in pediatric population [2]. In addition to being an uncommon diagnosis in the pediatric population, WE usually causes ocular motor abnormalities whereas fundus findings are atypical in adult or pediatric patients with WE. The treatment is thiamine repletion at earliest recognition of symptoms and signs. If not treated timely, it can lead to morbidity and mortality.

## Case presentation

A 17-year-old obese female presented with confusion and double vision. Her family reported that she had been dieting for the previous five months resulting in a 50-pound weight loss. Her diet mainly consisted of mashed potatoes with the recent addition of apple sauce in the last two weeks. In the week before admission, she had multiple presentations to an outside facility for evaluation of gastrointestinal issues like abdominal pain, headache, progressive confusion, and double vision. 

**Figure 1:**
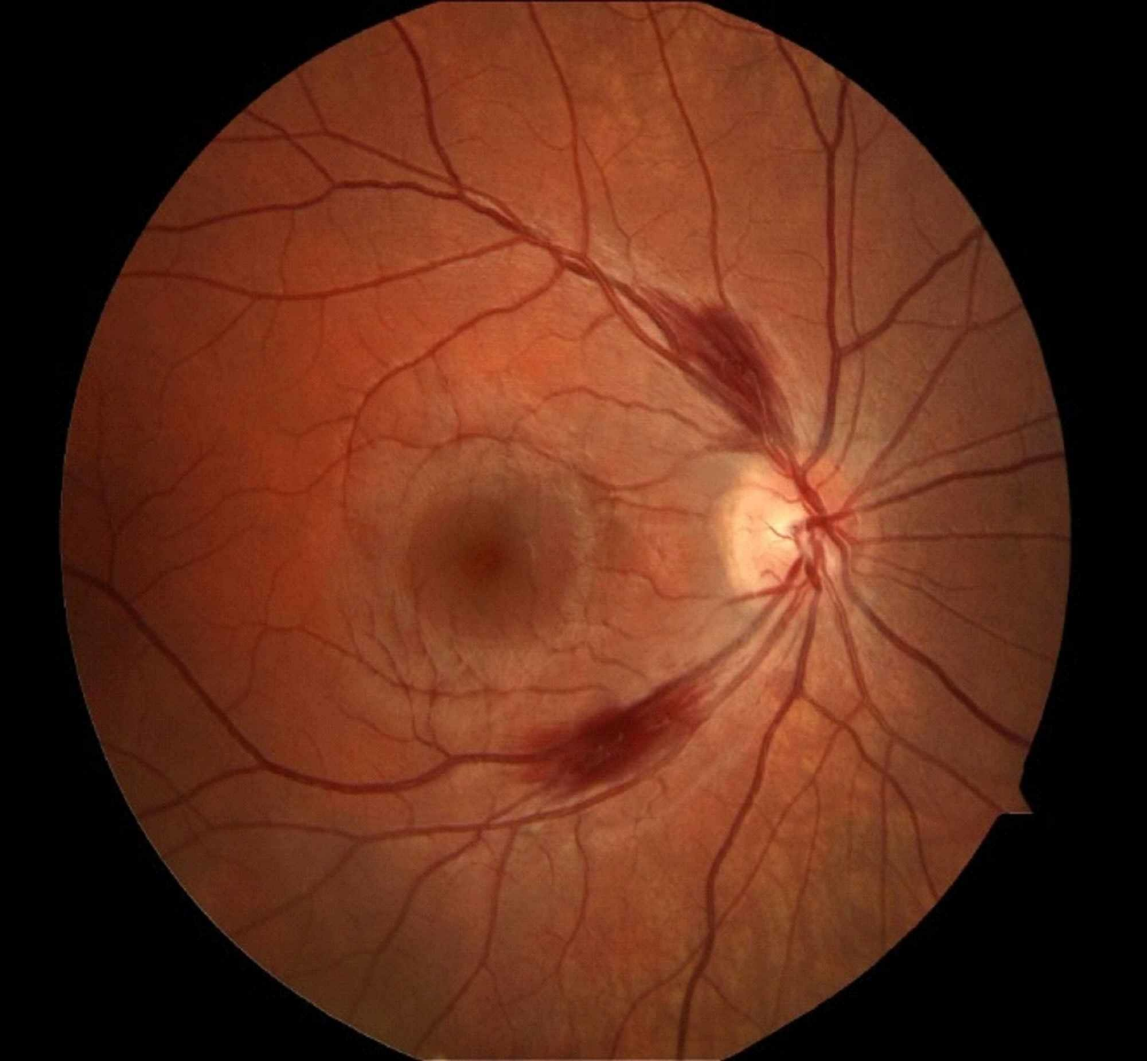
Fundus exam of right eye showing two peripapillary hemorrhages.

**Figure 2:**
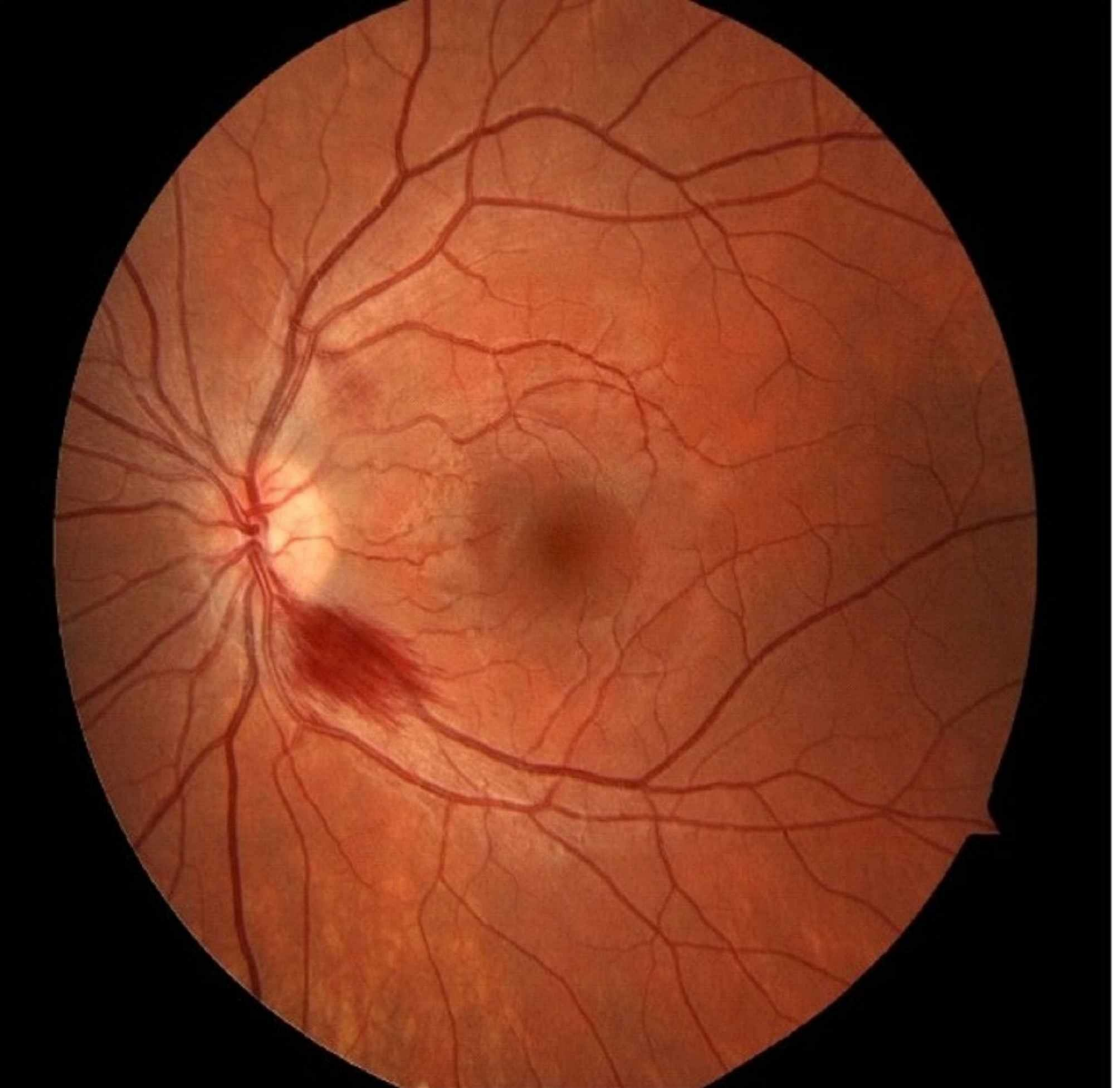
Fundus exam of left eye showing one peripapillary hemorrhage.

She denied nausea, vomiting, diarrhea, syncope episodes, but did acknowledge general fatigue. She denied intake of alcohol or other substances. Past medical or family history did not reveal any neurological or malabsorptive conditions. Neurology examination demonstrated an awake patient, oriented to person only, unable to answer the year, month, or date. Memory recall and repetition were impaired, but she had intact naming and speech fluency. Pupils were equal, round, and reactive to light and accommodation. Visual acuity was 20/20-3 bilaterally and the anterior chambers of the eyes were normal. No afferent pupillary defect noted. Extraocular movements demonstrated restriction in abduction and a slight decrease in adduction and depression bilaterally. While testing extraocular movements, vertical nystagmus was evident. On fundus examination, she had bilateral peripapillary retinal hemorrhages with intact optic disc margins (Figures 1, 2).

 

Motor and sensory examination were unremarkable. Coordination of finger to nose testing demonstrated dysmetria bilaterally. Reflexes were graded at 0 at the biceps, brachioradialis, triceps, patellar, and ankle tendons. Plantar response was flexor bilaterally. Gait testing demonstrated a wide-based ataxic gait.

**Figure 3:**
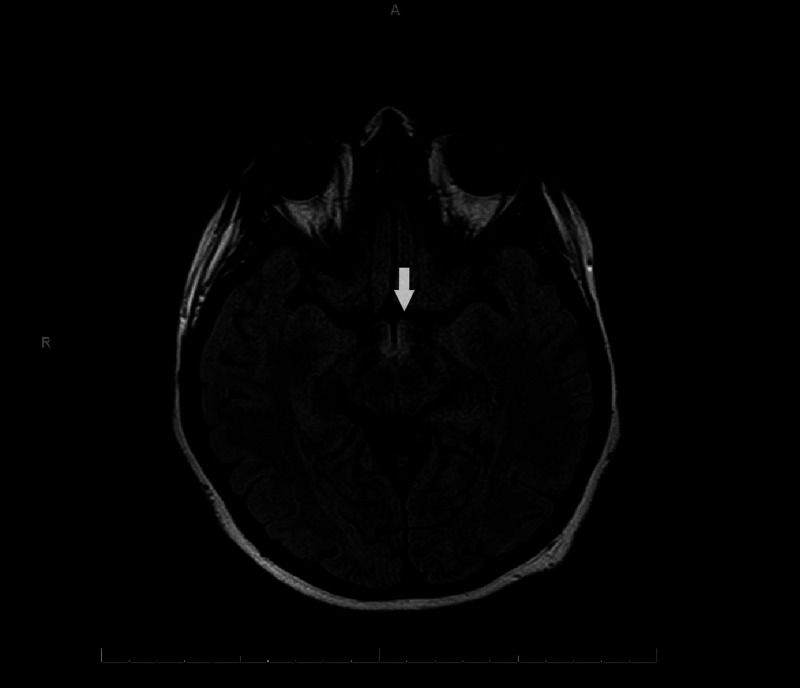
Axial T2 fluid-attenuated inversion recovery (FLAIR) image showing hyperintensity at mammillary bodies.

**Figure 4:**
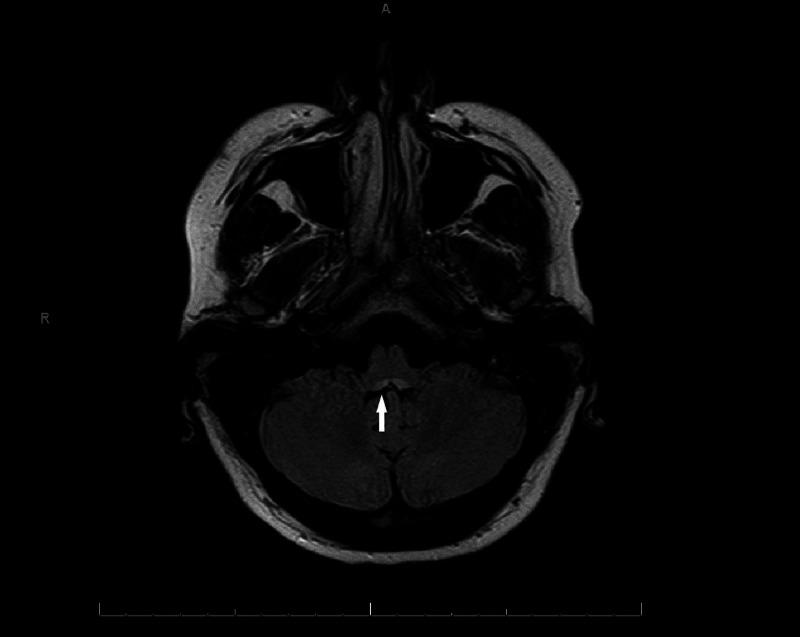
MRI axial T2 fluid-attenuated inversion recovery (FLAIR) brain image showing hyperintensity in posterior medulla.

**Figure 5:**
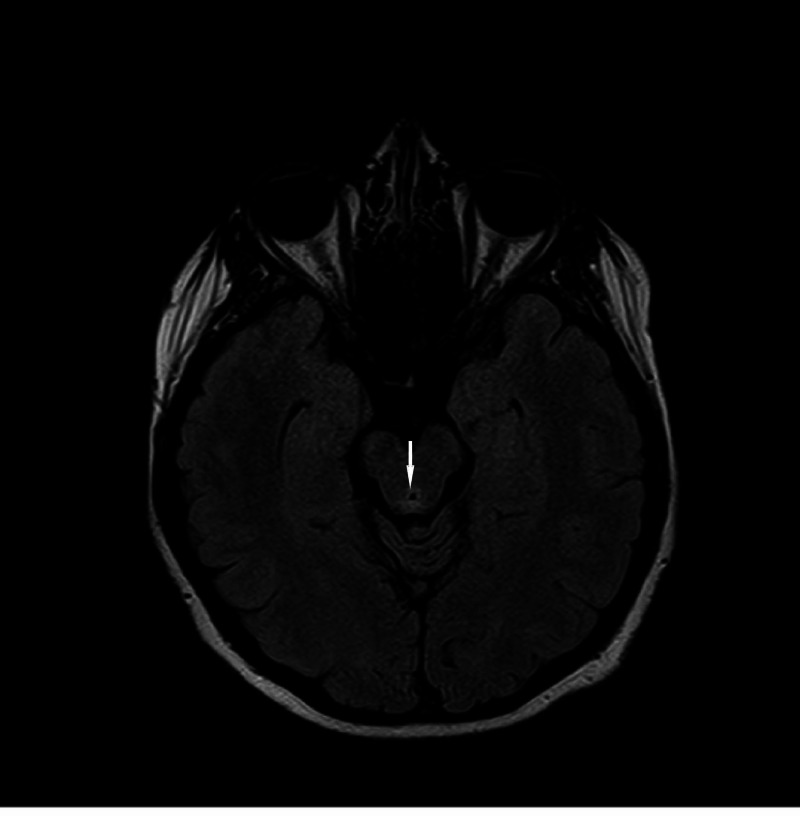
MRI axial T2 fluid-attenuated inversion recovery (FLAIR) brain image showing hyperintensity in periaqueductal gray area.

Complete blood count (CBC) and basic metabolic panel (BMP) were unremarkable. Her MRI brain demonstrated fluid-attenuated inversion recovery (FLAIR) hyperintensity in the periaqueductal gray matter, posterior medulla, mammillary bodies, and the medial thalami without edema or enhancement (Figures 3-5).

The remainder of the brain was normal in signal and morphology. Brain volume was normal for her age, and there was no abnormal enhancement.

These findings supported the diagnosis of WE, and she was placed on intravenous thiamine followed by oral supplementation. The patient showed gradual improvement.

## Discussion

We describe a case of a pediatric patient with confusion, ophthalmoplegia, areflexia, and ataxia following an attempt at weight loss with restricted food intake. Her clinical and radiographic findings were consistent with Wernicke syndrome secondary to thiamine deficiency.

WE is a neuropsychiatric condition first described by Dr. Carl Wernicke in late 19th century. Originally named “polioencephalitis hemorrhagica superioris” due to the autopsy findings, it was found to be linked to deficiency of thiamine [1]. Distinctive features of WE are paralysis of extraocular movements, gait abnormalities, and altered mental status. Commonly seen in adult patients with chronic alcoholism, it may also be seen with poor nutrition or impaired absorption. WE is considered an uncommon diagnosis in the pediatric population. If found in the pediatric population, malignancy is the most common etiology followed by patients on total parenteral nutrition [2].

Thiamine, a water-soluble vitamin, is required in many metabolic activities. Absorbed in the small intestine, the highest concentrations are found in skeletal muscle, liver, heart, and brain. There is limited tissue storage of thiamine, and levels are maintained only through continuous dietary intake [3].

Thiamine deficiency presents with two clinical phenotypes: Wernicke-Korsakoff syndrome and beriberi. Wernicke-Korsakoff syndrome is a term that denotes the encephalopathic syndromes. Wernicke is an acute entity and may respond to emergent intervention, whereas Korsakoff is a chronic entity.

Peripapillary hemorrhages on fundus examination, as seen in our patient, have been rarely seen in WE. It has been proposed that they are due to mitochondrial dysfunction secondary to thiamine deficiency, resulting in swelling and hemorrhage of the retinal nerve fiber layer. Rarely optic disc edema can also occur if the mitochondrial damage is severe and prolonged [4].

Due to the complaint of diplopia and findings of retinal hemorrhages in an obese patient, idiopathic intracranial hypertension was also on the differential. However, MRI and other clinical findings were suggestive of WE. Delaying thiamine supplementation and awaiting lumbar puncture procedure can be catastrophic for these patients. Thus, it is important to acknowledge that pediatric patients without any malignancy, who are on fad diets, like ours (eating mashed potatoes for the last five months), can have thiamine deficiency. Introduction of apple sauce (heavy glucose load) in the last two weeks may have acted as a precipitant in our patient as an increase in blood sugar, a high-energy state, increases the demand for thiamine. Patients who have deficiency of thiamine can quickly slide into WE when presented with intravenous glucose, as seen in our patient after a carbohydrate load. During the treatment, patients should be checked for other nutritional deficiencies as well as closely watched for refeeding syndrome [5,6].

## Conclusions

WE can present with retinal hemorrhages in the pediatric population. Supporting modalities for diagnosis like identifying classic clinical features and MRI will continue to be valuable but atypical presentation with fundus exam findings, like in our case, should also be taken into account. Thus, fundoscopy should be a part of the routine examination in WE-suspected patients. High-dose thiamine supplement must be started at the earliest to avoid chronic sequelae associated with this disease.
